# Antibacterial Action of a Condensed Tannin Extracted from Astringent Persimmon as a Component of Food Addictive Pancil PS-M on Oral Polymicrobial Biofilms

**DOI:** 10.1155/2016/5730748

**Published:** 2016-02-15

**Authors:** Kiyoshi Tomiyama, Yoshiharu Mukai, Masahiro Saito, Kiyoko Watanabe, Hidefumi Kumada, Tomotaro Nihei, Nobushiro Hamada, Toshio Teranaka

**Affiliations:** ^1^Department of Cariology and Restorative Dentistry, Graduate School of Dentistry, Kanagawa Dental University, 82 Inaoka-cho, Yokosuka, Kanagawa 238-8580, Japan; ^2^Institute of Oral Regenerative Medicine, Graduate School of Dentistry, Kanagawa Dental University, 82 Inaoka-cho, Yokosuka, Kanagawa 238-8580, Japan; ^3^Department of Restorative Dentistry, Division of Operative Dentistry, Graduate School of Dentistry, Tohoku University, 1-1 Seiryomachi, Sendai, Miyagi 980-8575, Japan; ^4^Department of Microbiology, Graduate School of Dentistry, Kanagawa Dental University, 82 Inaoka-cho, Yokosuka, Kanagawa 238-8580, Japan; ^5^Department of Dental Education, Graduate School of Dentistry, Kanagawa Dental University, 82 Inaoka-cho, Yokosuka, Kanagawa 238-8580, Japan; ^6^Department of Clinical Biomaterials, Graduate School of Dentistry, Kanagawa Dental University, 82 Inaoka-cho, Yokosuka, Kanagawa 238-8580, Japan

## Abstract

The purpose of this study was to evaluate the antibacterial activity against polymicrobial (PM) biofilms of a condensed tannin extracted from astringent persimmon (PS-M), which is contained in refreshing beverages commercially available in Japan. Salivary PM biofilms were formed anaerobically on glass coverslips for 24 and 72 h and were treated for 5 min with sterilized deionized water (DW), 0.05 and 0.2 wt% chlorhexidine digluconate (CHX), and 0.5–4.0 wt% PS-M solution. The colony forming units (CFU/mL) were determined and morphological changes of the biofilms were observed by scanning electron microscopy (SEM). The CFUs were lower in all PS-M and CHX groups compared to the DW group. PS-M exerted a dose-dependent effect. PS-M (1.53 × 10^7^) at a dose of 4.0 wt% had the same effect as 0.2 wt% CHX (2.03 × 10^7^), regardless of the culture period. SEM revealed the biofilm structures were considerably destroyed in the 4.0 wt% PS-M and 0.2 wt% CHX. These findings indicate that the antibacterial effects of PS-M, a naturally derived substance, are comparable to those of CHX. PS-M may keep the oral cavity clean and prevent dental caries and periodontal disease related to dental plaque, as well as systemic disease such as aspiration pneumonitis.

## 1. Introduction

In Japan, pneumonia is the third-most frequent cause of mortality, following malignant neoplasms and cardiovascular disease (the Ministry of Health Labour and Welfare's 2014 “Japan's Demographics”). A recent study reported that 70% of pneumonia cases in geriatric patients are related to misswallowing [1].

The Great East Japan Earthquake occurred on March 11, 2011. The Director General for Economic, Fiscal and Social Structure of Japanese government has reported that prolonged living in evacuation shelters will increase the number of patients with pneumonia and oral diseases. The number of patients from March 11 to April 10, 2011, was 2.7 times greater than that during the same period in 2010 (1223 versus 443, resp.). The prevalence of asthma, exacerbation of chronic obstructive pulmonary disease, and community-acquired pneumonia were also 2-3 times greater in 2011 than in 2010 (98 versus 32, 117 versus 46, and 443 versus 202, resp.) in all age groups in the same period as mentioned above. Half of the community-acquired pneumonia cases originated in evacuation shelters.

Aspiration pneumonia is caused by the bacteria that normally reside in the oral and nasal pharynx. Historically, aspiration pneumonia referred to an infection caused by less virulent bacteria, primarily oral pharyngeal anaerobes. It is now recognized that many common community-acquired and hospital-acquired pneumonia cases result from the aspiration of pathogens from the oral cavity or nasopharynx [2, 3]. The microorganisms that commonly cause this pneumonia, such as* Streptococcus pneumoniae*,* Haemophilus influenzae*,* Staphylococcus aureus*, and gram-negative bacteria are relatively virulent so that only a small inoculum is required to result in pneumonia [4, 5].

To prevent recurrent aspiration pneumonia, antibiotic therapy has been recommended, wherein normal inhabitants of the human oral cavity and drugs efficacious against the causal fungi are administered (the Japanese Respiratory Society and Nursing and Healthcare-Associated Pneumonia Guideline [6]). However, at present, there is no prominent antimicrobial therapy.

Because it has been difficult to culture and identify intraoral biofilms, which contain a wide variety of bacteria, there has been little progress in research on effective antibiotic therapy [7]. It has been reported that, following antibacterial treatment, biofilms with a large number of bacterial types behave differently from those with only a single or few types of bacteria [8]. Exterkate et al. [8] used stimulation saliva collected from subjects and developed a model that enables culture of polymicrobial (PM) biofilms comprising a large variety of intraoral bacteria outside the oral cavity. Since this model creates biofilms that are representative of those typically found within the oral cavity outside the oral cavity and contains a large number of bacterial types, it is a suitable model for high-throughput screening for investigating antibiotics effective for treatment of aspiration pneumonitis.

An ideal antibiotic should be safe and effective should not lead to resistant strains. Antibacterial effects of tannins have been recognized for a long time. In particular, condensed extract derived from green astringent persimmon fruit has strong protein regulatory and antibacterial effects [9]. Recent reports have demonstrated antibacterial effects of tannins on biofilm formation [10] and antibiofilm activities of tannins on* S. aureus* surface colonization [11, 12].

The purpose of the present study is to investigate the inhibitory effects of the above-mentioned tannin on intraoral biofilm formation by using PM biofilms consisting of multiple bacteria types found in oral microbiota. A portion of the results was presented at the 7th Annual Congress of the International Association for Dental Research Pan European Region (IADR/PER, 2014).

## 2. Materials and Methods

### 2.1. Antibiotic Agent

We used Pancil PS-M® (PS-M; Rilis Co., Ltd., Osaka, Japan), which contains 21.5 wt% of condensed tannin as the active ingredient which is extracted from green astringent persimmon fruit (*Diospyros kaki* Thunb.). It is also used as a food additive.  Table 1 shows the ingredients of PS-M. In this study, PS-M (0.5P, 0.7P, 1P, 2P, 3P, and 4P) was diluted to 0.5, 0.7, 1.0, 2.0, 3.0, and 4.0% (wt/vol), respectively, by adding sterilized deionized water (DW) and was used together with 0.05 and 0.2% (wt/vol) chlorhexidine digluconate (CHX; Corsodyl, GlaxoSmithKline, Brentford, London, UK), an effective antibiotic agent (Table 2). As 32 cases of anaphylaxic shock caused by 0.2–1% CHX have been reported in Japan in the last 21 years, only concentrations less than 0.05% are approved by the Ministry of Health, Labour and Welfare, Japan [13, 14]. DW was used as the control.

### 2.2. Specimens

Washed glass coverslips (*ϕ* 12 mm, thickness 0.15 mm, Menzel, Braunschweig, Germany) were used as a substrate for forming PM biofilms. Sterilization was performed in an autoclave (121°C, 20 minutes, and 2 atmospheres) after fixing the specimen on the lid dedicated to the high-throughput active attachment model [8].

### 2.3. Collecting Saliva

Saliva was collected from healthy adults with healthy dentition (without caries or periodontitis). The inclusion criteria were as follows: not having been given antibiotics or antibacterial agents for 3 months, not having brushed their teeth for the last 24 h, and not having had anything to eat or drink since 2 h before saliva collection. Subjects chewed paraffin wax (Parafilm M Barrier Films, Pechiney Plastic Packaging, Chicago, IL, USA), and the stimulated saliva was collected into a plastic container which was cooled down with ice. The saliva was kept at −80°C after being diluted into a 70 vol% solution with glycerol following filtration by glass wool. The study protocol was approved by the Institutional Review Board of Kanagawa Dental University (approval number 206).

### 2.4. PM Biofilms Formation

Retained saliva was diluted 50-fold with a semidefined medium (buffered McBain medium [15]; 2.5 g/L mucin, 2.0 g/L Bacto Peptone, 2.0 g/L trypticase peptone, 1.0 g/L yeast extract, 0.35 g/L NaCl, 0.2 g/L KCl, 0.2 g/L CaCl_2_, 1 mg/L hemin, 0.2 mg/L vitamin K_1_, 0.2% sucrose, and 50 mmol/L PIPES, pH 7.0). Medium containing 1.5 mL of saliva was injected into a 24-well plate; the glass coverslips that were fixed onto the dedicated lid were permeated and cultured at 37°C for 10 h in anaerobic conditions (10% CO_2_, 10% H_2_, and 80% N_2_) to form PM biofilms. Thereafter, they were soaked in the medium without saliva in the same manner and were cultured for 14 h. PM biofilm formation was stopped at this time in the 24-hour group. In the 72-hour group, culturing was continued by replacing the culture solution at cycles of 10 h and 14 h per day, and, at 72 h, PM biofilm formation was stopped. Glass coverslips with 24- or 72-hourPM biofilms were soaked into the 24-well plates with antibiotic agents or DW for 5 min at 22°C. To wash off various antibiotics, plates were soaked in wells into which 2 mL of cysteine peptone water (CPW) was added; then, the plates were shaken up and down 10 times. This process was repeated three times. Twelve glass coverslips were used for each group for the analysis of colony forming units (CFUs), and six glass coverslips were used for scanning electron microscopy (SEM).

### 2.5. Calculation of Viable Cell Counts following Treatment

**Figure 1:**
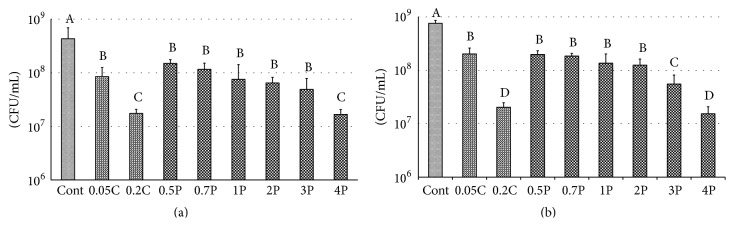
Total CFU counts of 24-hour (a) and 72-hour (b) PM biofilm after treatment. The CFU of 4P and 0.2C showed identical number of viable cells. Values with the same superscript letters are not significantly different between the groups at a *p* value of < 0.05. One-way ANOVA, Tukey's test (*n* = 12).

PM biofilms were collected into CPW from glass coverslips through ultrasound vibration (Transsonic T780, Elma Electric GmbH, Stuttgart, Germany), and a tube mixer (VTX-3500, LMS, Tokyo, Japan) was used to disperse the bacteria for 30 sec. Following serial dilution, a blood agar medium was smeared with the bacteria that were cultivated for 96 hours under anaerobic condition (10% CO_2_, 10% H_2_, 80% N_2_, and 37°C), and the CFUs were counted (Figure 1).

### 2.6. SEM

Specimens for SEM were prepared following the completion of antibiotic treatment by fixing them with 2% glutaraldehyde and 2% osmium and by performing gold vapor deposition following dehydration through serial alcohol dilution. SEM observation was performed under 20 kV condition (X-560; Hitachi, Tokyo, Japan) (*n* = 6).

### 2.7. pH Measurement of Spent Medium

pH was measured (9618-10D, F-71, Horiba, Kyoto, Japan) after stirring all replaced culture solutions for 10 sec using a vortex (tube mixer VTX-3500, LMS, Tokyo, Japan). Briefly, we measured the pH of all culture solutions at 10 and 24 h for the 24-hour culture test and at 10, 24, 34, 48, and 58 h for the 72-hour culture test.

### 2.8. Statistical Analysis

Differences in CFU were compared between the groups by one-way ANOVA followed by Tukey's test. All statistical analyses were performed using SPSS (version 10.11) with *p* < 0.05 defined as statistically significant. The group size for CFU counts was 12 for 18 experimental groups including 24 h and 72 h biofilm growth groups. In each lid, a water-treated control group was included.

## 3. Results

### 3.1. Live Bacterial Count following Antibiotic Treatment

The results of a live bacteria count following antimicrobial agent treatment of PM biofilms for 24 and 72 h are shown in Figures 1(a) and 1(b), respectively.

The CFU of 4P and 0.2C groups had an identical number of viable microorganisms. Significant differences were observed between the control and experimental groups regardless of the culture period. Values with the same superscript letters do not significantly differ among groups at *p* > 0.05.

Significantly lower CFU was observed in 24- and 72-hour biofilms in all treatment groups compared to the controls. PS-M reduced the bacterial count in the PM biofilms in a concentration-dependent manner. In 24- and 72-hour PM biofilms, suppression in CFU induced by in 0.5, 0.7, 1, and 2P was equivalent to that observed with 0.05C, and CFU of 4P was equivalent to that of 0.2C. In 72-hour PM biofilms, CFU in 3P was significantly lower compared to CFU in 2P. Moreover, in both 24- and 72-hour culture groups, 4P and 0.2C showed significantly lower bacterial counts compared to those in other groups. Antibacterial treatment resulted in a 97% reduction in the number of bacteria even in 72-hour culture PM biofilms, showing strong antibacterial properties (Table 2).

### 3.2. SEM Images of PM Biofilms

**Figure 2:**
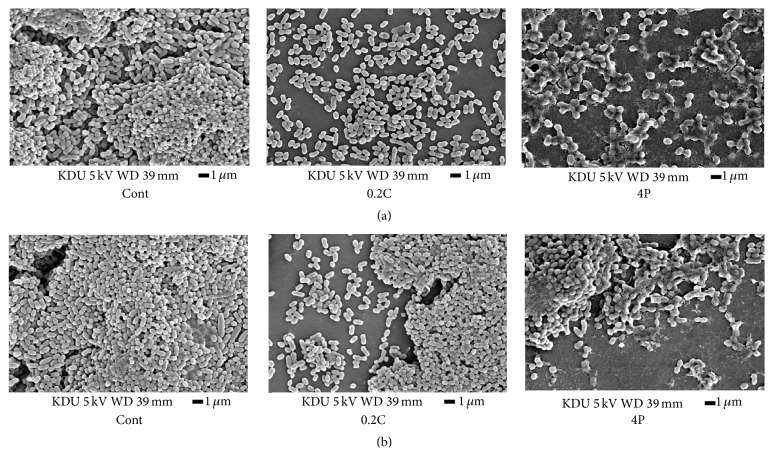
SEM images of 24-hour (a) and 72-hour (b) PM biofilms after treatment. 4P and 0.2C indicated suppression of bacterial aggregation comparing Cont.

SEM images of 24- and 72-hour cultured PM biofilms following 4P and 0.2C treatment showed the destruction of the PM biofilm structure and adherent destruction to substrate compared to controls (Figure 2). 4P and 0.2C groups displayed suppression of bacterial aggregation.

### 3.3. Spent Medium pH

**Figure 3:**
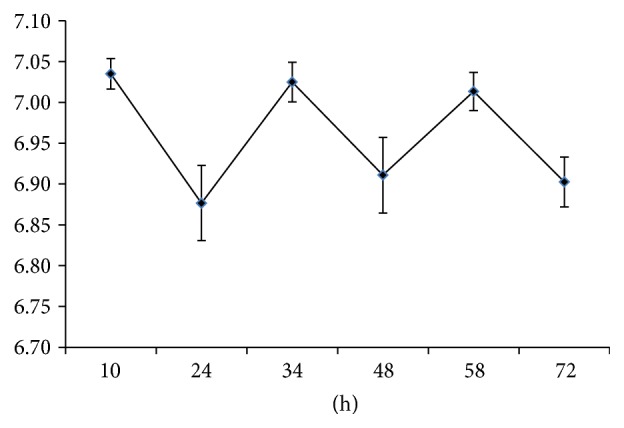
pH of spent medium. The pH of spent medium was shifted between 6.85 and 7.05 with the change of times.

The pH of replaced culture solutions in the control group remained at pH 7.05–6.85 (around neutral) during the culture period of 10–72 hours (Figure 3). There were no significant differences in pH between the groups at the same time periods.

## 4. Discussion

Based on the studies with medically relevant bacteria, it has been established that bacteria in biofilms are invariably less susceptible to antimicrobial agents than their planktonic counterparts [16]. Therefore, studies on agents that may be effective for treating plaque-related diseases should focus on organisms present in biofilms.

It is believed that intraoral biofilms consist of at least 800 types of bacteria [17, 18]. The PM biofilm used in the present study is a novel, high-throughput, and active attachment model. It is an effective method of forming biofilms consisting of multiple bacterial types similar to those typically found in the oral cavity under the same conditions; this is done by storing stimulated human saliva at −80°C following sterilized glycerin preparation and by defrosting and using it for each test [8]. In addition, the method can easily be implemented, and, therefore, the present biofilms consisting of multiple bacterial types are the most suitable biofilm model for the investigation of antibiotic agents for preventing systemic diseases, such as aspiration pneumonitis.

In the present study, to investigate the concentration-dependent antibacterial effects of astringent persimmon tannin, we compared the effects of 0.5, 0.7, 1, 2, 3, and 4 wt% Pancil PS-M to those of 0.05 and 0.2% CHX. The results showed that Pancil PS-M solutions have concentration-dependent antibacterial effects on PM biofilms. Furthermore, their effects were the same regardless of their culture period. In other words, they were observed to have similar trend for biofilms cultured for the two different time periods of 24 or 72 hours. In particular, 4P in the same way as 0.2C significantly suppressed the PM biofilm live bacterial count compared to other groups, showing a higher live bacterial count reduction rate. Additionally, we configured a group that only contained trehalose and sodium carbonate at the same concentration with 4P group, and its antibiofilm effect was investigated to 24-hour biofilm. As a result, there was no significant difference of CFU compared to the control (DW) group (data was not shown). Therefore, we can exclude any effects derived from the other chemical components of the Pancil PS-M.

Condensed tannin derived from astringent persimmon has been shown to have a strong protein coagulation action and antibacterial effect. Catechols derived from* Diospyros kaki* Thunb. have significant antibacterial effects against* C. difficile* and* E. coli* [19]. Furthermore, persimmon tannin has been shown to exhibit strong antiviral effects against a wider range of viruses compared with tannins derived from green tea, acacia, and gallnuts [20]. In the present study, we used tannin extracted from the unripened fruit of* Diospyros kaki* Thunb.

One reason for the astringent persimmon tannin-induced suppression of saliva-derived biofilm formed on glass surfaces is hydrophobicity, which plays an important role in the adherence of bacterial cell bodies to the surfaces of teeth [21]. For example, it is believed that hydrophobicity of* S. mutans* is primarily mediated by bacterial cell surface proteins [22]. A previous study has reported that* S. mutans* and* S. sanguinis* cannot adhere to hydroxyapatite if their hydrophobicity is lost [23]. Although it has been demonstrated that polyphenols in Oolong tea prevent* S. mutans* from adhering to the tooth surface by reducing its bacterial cell body hydrophobicity [24], there have been no reports on its effects on biofilms consisting of multiple bacteria. In our study, SEM demonstrated differences in bacterial cells between those subjected to CHX processing and those subjected to astringent persimmon tannin processing. In other words, bacterial cell formation changed following astringent persimmon tannin processing. The reason for this may be that CHX has antibacterial properties which have an impact on the permeability of bacterial cells but not on bacterial cell formation. However, astringent persimmon tannin may have an impact on both bacterial cell formation and live bacterial count by destroying bacterial cell walls. Although it is assumed that the inside of PM biofilms cultured for 72 h is highly anaerobic compared to that of PM biofilms cultured for 24 h, we did not detect differences in bacterial formation. In the present study, the pH of cultured solutions was maintained approximately neutral at all times by adding a buffer agent. Therefore, we considered that bacteria within the biofilms reproduced a condition of early-stage plaque adhesion.

*Diospyros kaki* Thunb. has effects not only on bacteria but also on the norovirus. Kamimoto et al. [25] have reported that persimmon extract has remarkable effects on human norovirus (NoV GII. 4). Furthermore, in a clinical study, Gato et al. [26] have demonstrated that tannin derived from astringent persimmon reduces cholesterol and blood glucose levels. Another study has reported that people with a large amount of biofilms formed on the tongue have higher acetaldehyde levels, which increases the risk of esophageal and pharyngeal cancers [27, 28]. Taken together, these findings suggest that astringent persimmon tannin is an effective agent that maintains and improves health not only in the oral cavity but also in the entire body.

CHX is an effective antibacterial agent with a wide antibacterial spectrum that ranges from bacteria to fungi, high safety including low irritation of the skin and mucosa, a small degree of reduced drug effects due to biological components, such as blood and body fluids, and sustained drug effects following biological disinfection based on tissue type. CHX was developed approximately 50 years ago and is still widely used in the clinical settings worldwide. However, in recent years, it has been demonstrated that bacterial strains resistant to the presently used antibiotics include* S. aureus*,* Pseudomonas aeruginosa*,* Burkholderia cepacia*, and* Serratia marcescens*. This finding indicates the emergence of bacteria resistant to other antibacterial agents and represents a growing problem in the prevention of hospital infections [29]. 0.2% CHX is generally sold in Europe and USA as the maximum concentration for mouthwash agents. Exterkate et al. [30] activated 0.2% CHX at room temperature for 5 min for salivary PM biofilms cultured for 24 h and reported that this preparation significantly reduced the production of CFUs and lactic acid. Hope and Wilson [21] activated 0.05 and 0.2% CHX for 5 min for multispecies biofilms formed within constant-depth film fermenters and investigated them using confocal laser scanning microscopy. They reported that the impact of 0.05% CHX processing on biofilms could not be detected and that a clear impact of 0.2% CHX processing on biofilms structure was detected.

Therefore, we chose the exposure time of 5 min for both chlorhexidine and PS-M.

The results of the present study also showed that 0.2% CHX has a significant impact on biofilms. However, it is possible that the efficacy of CHX in oral care is now being undermined. A meta-analysis by Alhazzani et al. has demonstrated that the use of CHX significantly reduces the effects of tooth brushing [22].

As mentioned above, it has been shown that PS-M containing condensed tannin derived from astringent persimmons not only has antibacterial properties but also has antiviral properties and is effective for circulatory and metabolic diseases. It has been suggested that it has a high potential in prevention of dental diseases and aspiration pneumonitis in geriatric patients and recovering patients when it is added to mouthwash and toothpaste.

## 5. Conclusion

We have shown that 4 wt% PS-M containing condensed tannin derived from astringent persimmons has antibacterial actions against biofilms containing multiple bacteria and that these effects are as strong as those of 0.2 wt% CHX. These findings strongly suggest that PS-M may be helpful in improving the intraoral environment.

## Figures and Tables

**Table 1:** Components of Pancil PS-M.

Condensed tannin (wt%)	21.5
Trehalose (wt%)	66.4
Sodium carbonate (wt%)	6.6
Water (wt%)	4.5
Others (wt%)	
Protein, lipid, polysaccharide, ash	1.0

**Table 2:** Effects of a single treatment with PS-M and chlorhexidine digluconate on the total CFU counts of 24- and 72-hour polymicrobial biofilms.

Group	Reduction rate (%)	
	24 h	72 h
Cont	—	—
0.05C	80	73
0.2C	95	97
0.5P	65	73
0.7P	73	75
1P	82	82
2P	84	83
3P	88	92
4P	96	97
